# Differential Zika Virus Infection of Testicular Cell Lines

**DOI:** 10.3390/v11010042

**Published:** 2019-01-09

**Authors:** Luwanika Mlera, Marshall E. Bloom

**Affiliations:** Biology of Vector-Borne Viruses Section, Laboratory of Virology, Rocky Mountain Laboratories, NIAID/NIH, Hamilton, MT 59840, USA; Luwanika.Mlera@gmail.com

**Keywords:** flavivirus, Zika virus, viral persistence, testicular cells, testes

## Abstract

Background: Zika virus is a mosquito-borne flavivirus responsible for recent outbreaks of epidemic proportions in Latin America. Sexual transmission of the virus has been reported in 13 countries and may be an important route of infection. Sexual transmission of ZIKV has mostly been male-to-female, and persistence of viral RNA in semen for up to 370 days has been recorded. The susceptibility to ZIKV of different testicular cell types merits investigation. Methods: We infected primary Sertoli cells, a primary testicular fibroblast Hs1.Tes, and 2 seminoma cell lines SEM-1 and TCam-2 cells with ZIKV Paraiba and the prototype ZIKV MR766 to evaluate their susceptibility and to look for viral persistence. A human neuroblastoma cell line SK-N-SH served as a control cell type. Results: Both virus strains were able to replicate in all cell lines tested, but ZIKV MR766 attained higher titers. Initiation of viral persistence by ZIKV Paraiba was observed in Sertoli, Hs1.Tes, SEM-1 and TCam-2 cells, but was of limited duration due to delayed cell death. ZIKV MR766 persisted only in Hs1.Tes and Sertoli cells, and persistence was also limited. In contrast, SK-N-SH cells were killed by both ZIKV MR766 and ZIKV Paraiba and persistence could not be established in these cells. Conclusions: ZIKV prototype strain MR766 and the clinically relevant Paraiba strain replicated in several testicular cell types. Persistence of ZIKV MR766 was only observed in Hs1.Tes and Sertoli cells, but the persistence did not last more than 3 or 4 passages, respectively. ZIKV Paraiba persisted in TCam-2, Hs1.Tes, Sertoli and SEM-1 cells for up to 5 passages, depending on cell type. TCam-2 cells appeared to clear persistent infection by ZIKV Paraiba.

## 1. Introduction

Zika virus (ZIKV) is a mosquito-borne flavivirus originally described in captive *Macaca mulatta* monkeys in Uganda [1]. ZIKV recently caused an outbreak of epidemic proportions in Latin American countries and was associated with devastating microcephaly in neonates that contracted the infection in utero [2]. Other complications of ZIKV are varied and include Guillian Barre syndrome [3,4,5,6].

Although ZIKV is primarily transmitted by *Aedes* mosquito bites, sexual transmission is now well-documented. The first description of sexual transmission is probably that of 2 American scientists who were bitten by *Aedes* mosquitoes while working in Senegal in 2008 [7]. The male transmitted ZIKV to his wife and she presented clinical signs of disease consistent with ZIKV infection [7]. Additional recent reports described infection in partners following travel to outbreak regions [8,9]. An interesting example is that of an asymptomatic French couple who were only diagnosed when they sought assisted reproductive health services after returning from the French island of Martinique [8]. Most of the sexual transmission cases reported have been male-to-female, but a suspected female-to-male case has been reported [10]. To date, 13 countries have documented sexual transmission of ZIKV [11]. In the US in 2016, 47/5168 ZIKV cases were attributed to sexual transmission [12], whereas 8/451 cases could have been sexually transmitted in 2017 [13]. Thus, sexual transmission may be an important route of acquiring infection although it would be difficult to assess such transmission in the face of a large vector-borne outbreak [14].

The testes are male organs that contain germ cells which differentiate into mature spermatozoa. Sertoli cells are interspaced between germinal epithelial cells and provide support for the germ cells. Leydig cells are irregularly shaped interstitial cells that produce the hormone testosterone. Sexual transmission of ZIKV by males and the presence of virus in semen suggests that cells in the male genitourinary tract are infected [15]. Animal studies have also shown that the testes are infected with various consequences, including testicular atrophy with implications in male fertility [16,17]. Virus was reported to be mainly in the interstitial Leydig cells and Sertoli cells, but this varied from study to study [16,18,19]. Govero and colleagues showed that Sertoli cells detached from the basement membrane and that there was a decline in the germ cell population in ZIKV infected mice [17]. Thus, the different cells in the testes may play different roles in harboring virus for transmission or pathogenesis, which leads to the destruction of organ integrity.

In this paper, we infected several human testicular cells lines to evaluate the extent to which the cells permitted ZIKV replication in vitro; primary Sertoli cells, a primary testicular fibroblast Hs1.Tes and the 2 seminoma cell lines SEM-1 and TCam-2. The infection in the testicular cell lines was compared to infection in a human neuroblastoma cell line SK-N-SH. We were also interested in determining if ZIKV would persist in any of these cell lines. Our results showed that ZIKV differentially infected the testicular cell lines tested and could persist in some cells in a strain-dependent manner. Delayed apoptotic cell death was observed during viral persistence, thus limiting duration of persistence to 5 passages at most.

## 2. Materials and Methods

### 2.1. Viruses and Cells

The Ugandan ZIKV strain MR766 were generously provided by Dr. Stephen Whitehead (Laboratory of Infectious Disease, NIAID/NIH). The Brazilian ZIKV Paraiba was isolated by Dr. Pedro F.C Vasconcelos, Instituto Evandro Chagas, Brazil and it was a kind gift from Dr. Stephen Whitehead (Laboratory of Infectious Disease, NIAID/NIH). Virus stocks were prepared by infecting Vero (ATCC) cells and harvesting the supernatants 3 days post infection. Virus in the supernatants was semi-purified by ultracentrifugation over a 20% sucrose solution, followed by quantification using a plaque assay on Vero cells. 

The neuroblastoma SK-N-SH cell line [20] was purchased from ATCC and maintained in antibiotic-free Eagle’s minimum essential medium (EMEM; Gibco, Hampton, NH, USA) containing 10% fetal bovine serum (FBS). Fibroblast Hs1.Tes cells (ATCC) and the TCam-2 seminoma cell line (a kind gift from Dr. Constantine Stratakis, NICHD/NIH) were maintained in Dulbecco’s modified Eagle’s medium (DMEM) supplemented with 10% FBS and 1× antibiotic-antimycotic (Gibco). Doubling time for the TCam-2 seminoma cell line in culture is approximately 50 h. Sertoli cells (Lonza, Basel, Switzerland) were grown in DMEM/F12 medium with 10% FBS and penicillin/streptomycin (Gibco) and the cells grow to confluence in 7–10 days when seeded at 450–500 cells/cm^2^. SEM-1 seminoma cells were a kind gift from Dr. Alan Epstein (USC Keck School of Medicine, Los Angeles, CA, USA), and they were maintained in RPMI 1640 (Gibco/ThermoFischer) supplemented with 10% FBS and penicillin/streptomycin. SEM-1 cell doubling time is 50 h.

### 2.2. ZIKV Infection

Two million cells were seeded in 75 cm^2^ flasks a day before the infection and allowed to grow at 37 °C and 5% CO_2_. ZIKV infections with either MR766 or Paraiba strains were performed at a multiplicity of infection of 0.1 with adsorption at 37 °C and rocking for 1 h. All infections were performed in triplicate (biologically independent replicates). The inoculum was removed, and cells were washed 3 times with phosphate buffered saline (PBS; Gibco). Twenty mL of the appropriate cell culture medium was added prior to incubation at 37 °C and 5% CO_2_. Supernatant aliquots were removed daily for 7 days and stored at −80 °C until virus titration. The infected cells were also microscopically observed daily for the development of cytopathic effect (CPE). At 7 dpi, intact cell monolayers were washed twice in PBS, trypsinized and passaged at 1:10 in new flasks with fresh culture media.

### 2.3. ZIKV Titration by Immunofocus Assay

Supernatants from infected cultures were harvested at different time points post infection. Serial 10-fold dilutions were carried out and 250 µL of each dilution was plated onto confluent Vero cells (ATCC) in 12-well plates. Each dilution was plated in duplicate. ZIKV was adsorbed for 1 h at 37 °C with rocking, followed by washing twice with PBS. The infected monolayer was overlaid with DMEM containing 0.8% methylcellulose, 2% FBA and antibiotics. Plates were incubated at 37 °C for 3 days after which the overlay was removed, and the cells were washed with PBS twice. The cells were fixed with 100% methanol for 15 min, washed twice with PBS and probed with an anti-ZIKV E antibody (BioFront Technologies, Tallahassee, FL, USA) at a 1:1000 dilution. Cells in the primary antibody were incubated at 37 °C for 1 h. Following primary antibody incubation, cells were washed twice with PBS and an anti-mouse secondary antibody (Dako, Santa Clara, CA, USA) was added at a 1:1000 dilution and incubated with the cells at 37 °C for 1 h. Next, the cells were washed twice with PBS followed by development of immunofoci with a diaminobenzidine/peroxide substrate (Sigma-Aldrich, St. Louis, MO, USA) and enumeration.

### 2.4. Monolayer Staining with Giemsa Stain

To visualize the cytopathic effect of ZIKV on infected cell monolayers, cells were washed twice in PBS and fixed with 4% paraformaldehyde (PFA) for 10 min at room temperature. The PFA was aspirated followed by washing twice with PBS. Cells were stained with a 1:5 Giemsa stain for 30 min. The stain was removed, and cells were washed twice with PBS and imaged using an AxioVert.A1 microscope equipped with Zeiss Axiocam 503 monochromatic camera.

### 2.5. Immunofluorescence Microscopy

ZIKV-infected Sertoli cells at P1 were plated into a 4-well chamber slide at 1 × 10^4^ cells/well and allowed to attach overnight at 37 °C in 5% CO_2_. The medium was aspirated, and cells were washed twice with PBS. The cells were fixed with 4% paraformaldehyde/5% sucrose in PBS for 10 min. The fixed cells were permeabilized with 0.1% Triton X/4% PFA in PBS for 10 min with shaking. Aldehydes were quenched using 50 mM glycine for 10 min. Blocking was done with 2% bovine serum albumin for 1 h. Cells were probed with a mouse anti-ZIKV E monoclonal antibody (BioFront Technologies) at 1:1000 dilution, and a rabbit anti-cleaved caspase 3 (BD Biosciences, San Jose, CA, USA) at 1:1000 dilution. The primary antibodies were detected with anti-mouse (conjugated with Alexa Flour 647) and Alexa Flour 488-conjugated anti-rabbit antibodies (Invitrogen, Carlsbad, CA, USA). Images were captured using a Laser Scanning Microscope (LSM) 710 (Zeiss, Oberkochen, Germany) at 40× magnification.

## 3. Results

### 3.1. ZIKV Infection of the Fibroblast Hs1.Tes Cell Line

**Figure 1:**
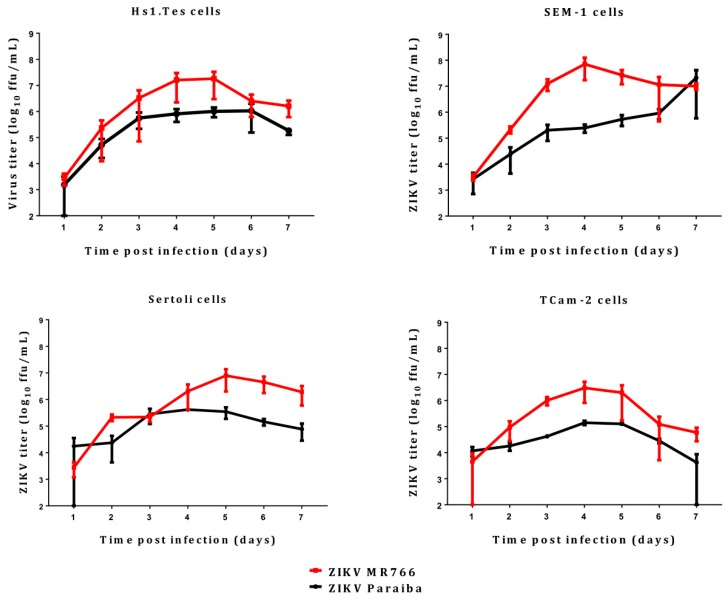
Replication kinetics of ZIKV MR766 and Paraiba over the course of 7 days. Error bars represent standard deviation (SD) from the mean for 3 independent replicates.

**Figure 2:**
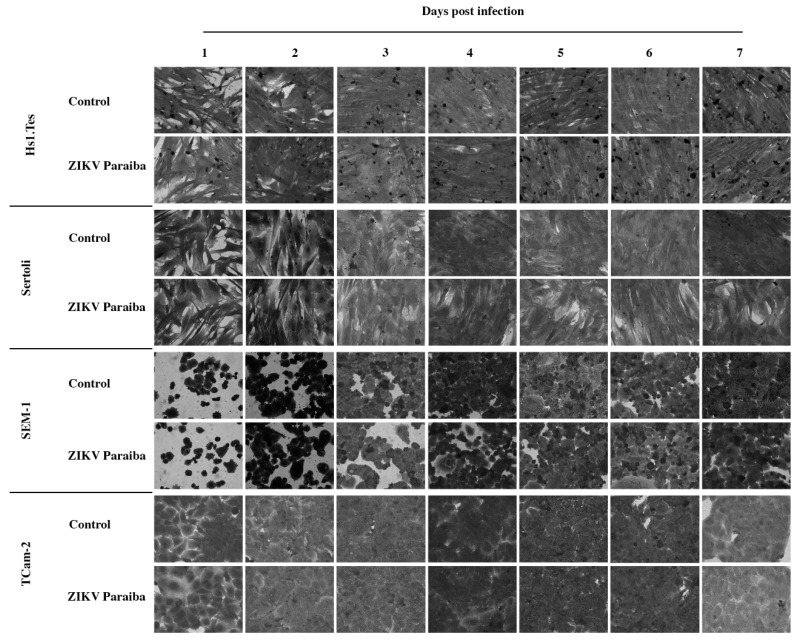
Microscopic evaluation of testicular cell lines infected with ZIKV Paraiba. No obvious cytopathic effect was observed in all testicular cell lines infected with ZIKV Paraiba. Images were captured at a magnification of 400×.

**Figure 3:**
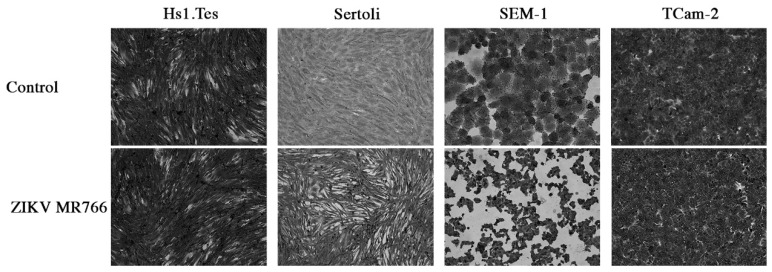
Cytopathic effect of ZIKV MR766 on testicular cell lines at 7 dpi. We noted that ZIKV MR766-infected Sertoli and SEM-1 cells appeared smaller than uninfected controls. TCam-2 and Hs1.Tes cells did show any CPE at 7 dpi. Images were obtained at a magnification of 400×.

The Hs1.Tes cell line is a fibroblast which represents testicular connective tissue. Both ZIKV MR766 and Paraiba strains were able to infect this cell line in culture, and ZIKV MR766 replicated to higher titers than ZIKV Paraiba (Figure 1). The ZIKV MR766 titer peaked to 1.8 × 10^7^ ffu/mL at 4 dpi, whereas ZIKV Paraiba titers peaked to 1.0 × 10^6^ at 5 dpi (Figure 1). Infection of Hs1.Tes cells with either ZIKV Paraiba or ZIKV MR766 did not result in any obvious cytopathic effect (CPE) by 7 dpi (Figure 2 and Figure 3).

**Figure 4:**
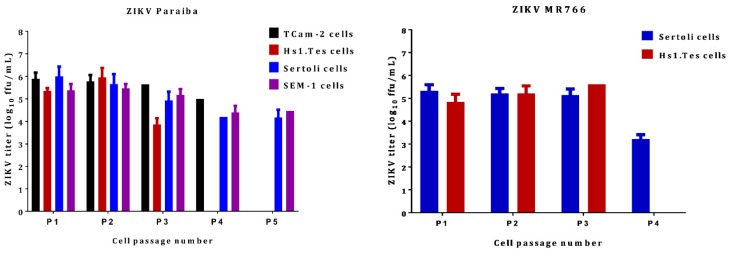
ZIKV titers in persistently infected testicular cell lines. The clinical isolate ZIKV Paraiba was able to persist in all cell lines tested for up to 5 passages (P1 through to P5), depending on cell line. ZIKV MR766 was only able to persist in Sertoli and Hs1.Tes cells. Virus titration was performed using supernatants collected at the end of each 7-day period and each data point represents an average of 3 biological replicates. Error bars represent standard deviation from the mean. Each passage was done after 7 days by washing the monolayer twice with PBS, trypsinizing the cells and seeding into new flasks with fresh culture medium at 1:10.

**Figure 5:**
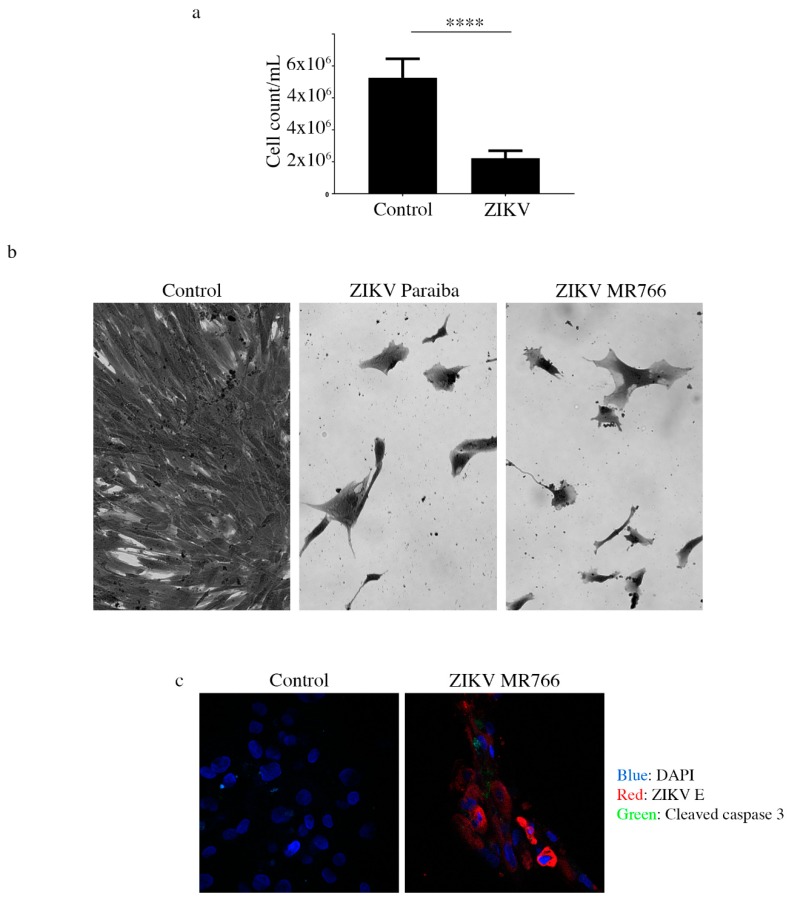
Analysis of continued cell death in ZIKV-infected testicular cells. (**a**) Cell count graph showing that there were 2-times more cells in the uninfected Hs1.Tes control, compared to ZIKV Paraiba-infected Hs1.Tes cells at P1. For both control and ZIKV-infected cells, the count was done after 7 days of cell passage. ****, *p* < 0.0001 (unpaired *t*-test). (**b**) Microscopic images showing loss of the Hs1.Tes monolayer in ZIKV-infected cells at P3. The morphology of infected cells at this time point appeared grossly aberrant and pleomorphic when compared to that of uninfected control cells. Cells were imaged at a magnification of 400×. (**c**) Confocal microscopy images showing cleaved caspase 3 in some ZIKV-infected Sertoli cells at P1. Not all ZIKV E protein-expressing cells stained positive for cleaved caspase 3, supporting the notion that cell death in persistently infected cells was progressive. Cells were imaged at a magnification of 400×.

To determine if ZIKV could persist in Hs1.Tes fibroblast cell line, we passaged the ZIKV infected cells after 7 dpi. Infectious ZIKV Paraiba and ZIKV MR766 was recovered from the Hs1.Tes supernatants at each passage for 3 passages, suggesting that ZIKV could persist in these cells (Figure 4). However, we observed a decline in the cell population upon passage each, suggesting delayed and slowly progressive cell death and a 4th passage was not possible (Figure 5a,b). Thus, the duration of ZIKV persistence in Hs1.Tes cells was limited by continued cell death.

### 3.2. ZIKV Infection of Sertoli Cells

Sertoli cells are testicular cells, which support the germ cells. In culture, we observed that these cells were also permissive to infection by both ZIKV Paraiba and MR766 strains, which replicated to maximal titers at 4 and 5 dpi, respectively (Figure 1). The ZIKV Paraiba titers did not proceed beyond 10^6^ ffu/mL for 7 dpi, but MR766 titers neared 10^7^ ffu/mL at 5 dpi. Similar to Hs1.Tes cells, infection of Sertoli cells with ZIKV Paraiba did not cause an observable cytopathic effect (CPE) by 7 dpi (Figure 2), but ZIKV MR766 caused minimal CPE which was observed at 7 dpi (Figure 3).

After 7 dpi, we passaged Sertoli cells infected with either ZIKV Paraiba or ZIKV MR766 and observed that ZIKV Paraiba persistently infected Sertoli cells for up to 5 passages before the delayed cell death, like that observed in Hs1.Tes cells, rendered the cells unfit for a 6th passage. Detection of cleaved caspase 3 by immunofluorescence (Figure 5c) suggested that cell death was via apoptosis. We also observed that not all ZIKV-infected cells stained positive for cleaved caspase 3 (Figure 5c). The progressive cell death was accompanied by a decline in the ZIKV Paraiba titers from 1.0 × 10^6^ ffu/mL at passage 1, to 1.5 × 10^4^ ffu/mL at passage 5 (Figure 4). ZIKV MR766 infection persisted in Sertoli cells for only 4 passages and the viral titers were constant at ~10^5^ ffu/mL from passage 1 to passage 3, but the titer declined to 1.6 × 10^4^ at the 4th passage (Figure 4).

### 3.3. ZIKV Infection of Seminoma Cell Lines

TCam-2 cells are a germ cell seminoma cell line, and SEM-1 cells are a testicular cell line which is an intermediate between a non-seminoma and a true seminoma [21,22]. Compared to all cell lines tested, SEM-1 seminoma cells supported the highest titers of both ZIKV MR766 and ZIKV Paraiba replication (Figure 1). The ZIKV MR766 titer peaked to 7.1 × 10^7^ at 4 dpi, whereas the peak ZIKV Paraiba titer was attained at 7 dpi (Figure 1). Interestingly, the ZIKV replication graph in SEM-1 cells suggested that ZIKV Paraiba was still replicating upwards of the peak attained at 7 dpi. No obvious CPE was observed in ZIKV Paraiba-infected SEM-1 cells (Figure 2), but ZIKV MR766 caused notable cell death by 7 dpi and the infected cells appeared smaller in comparison to uninfected controls (Figure 3). Upon a single passage, ZIKV MR766 infection induced a lytic crisis in SEM-1 seminoma cells. The culture of ZIKV MR766 infected cells did not recover, and thus, a persistent ZIKV MR76 6 infection of SEM-1 cells could not be established. However, a persistent ZIKV Paraiba infection was maintained in SEM-1 cells up to 5 passages (Figure 4).

In TCam-2 seminoma cells, ZIKV titers peaked at 4 dpi, with the MR766 strain at 3 × 10^6^ ffu/mL and the Paraiba strain at 1.4 × 10^5^ ffu/mL (Figure 1). By 7 dpi, ZIKV MR766 and ZIKV Paraiba titers declined to 6 × 10^4^ ffu/mL and 4 × 10^3^ ffu/mL, respectively. Interestingly, both ZIKV strains did not cause any CPE in TCam-2 cells by 7 dpi (Figure 2 and Figure 3). Although ZIKV Paraiba persisted in TCam-2 cells for 4 passages, ZIKV MR766 killed the cells upon passage into P1, suggesting a strain-dependent delayed cell death mechanism.

### 3.4. ZIKV Infection in a Human Neuroblastoma SK-N-SH Cell Line

**Figure 6:**
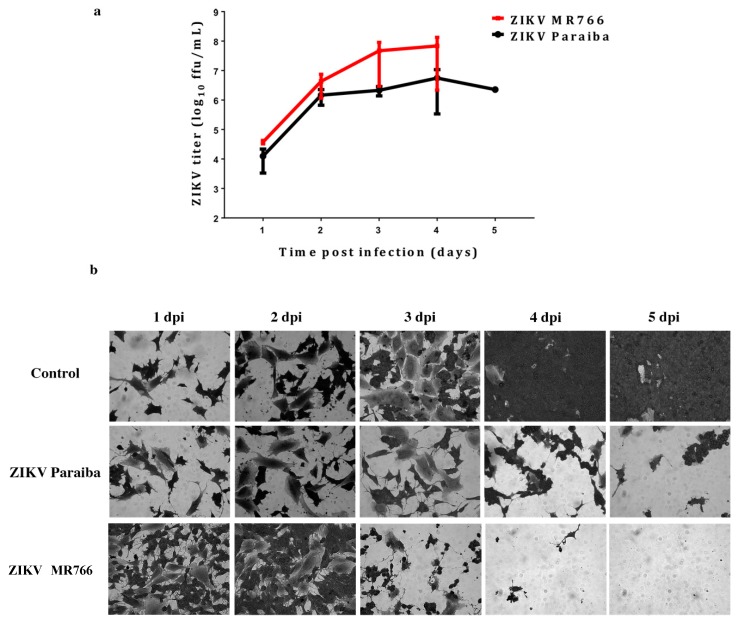
ZIKV infection in a human neuroblastoma SK-N-SH cell line. (**a**) Replication kinetics of ZIKV MR766 and Paraiba strains in SK-N-SH cells. Error bars represent SD from the mean for 3 independent experiments. (**b**) Cell death in SK-N-SH cell monolayers infected with ZIKV Paraiba or ZIKV MR766. ZIKV MR766 was more aggressive at killing SK-N-SH cells. Images of the SK-N-SH monolayer were acquired at a magnification of 400×.

Our laboratory has previously reported infection of the human neuroblastoma SK-N-SH cell line [23]. We used the SK-N-SH cells to compare ZIKV infection in testicular cells lines, and to determine if ZIKV would also persist in these cells. ZIKV MR766 replicated to higher titers than ZIKV Paraiba (Fig 6a) as expected [23]. Infection of SK-N-SH cells with either ZIKV MR766 or ZIKV Paraiba resulted in extensive CPE by day 4 or 5, respectively (Figure 6b). Following the lytic crises, the few remaining cells (Figure 6b) died a few days after media replenishment. Thus, no persistent ZIKV infection of SK-N-SH cells could be established.

## 4. Discussion

ZIKV has recently been shown to cause devastating effects in neonates following in utero infection [2,24]. In adults, sexual transmission has been well documented and was associated with testicular atrophy and male sterility [16,17,25,26]. A few reports have subsequently reported infection of testicular cells lines, particularly Sertoli cells [27,28,29]. In order to further evaluate these reports, we infected 4 testicular cell lines representing connective tissue (Hs1.Tes cells) and germ cells (SEM-1 and TCam-2 cells) as well as Sertoli cells with the prototype ZIKV MR766 and a clinical isolate ZIKV Paraiba to determine the extent to which the viruses would persist in these cells. 

All the cells we tested were permissive to both ZIKV MR766 and ZIKV Paraiba, but the former replicated to higher titers than the clinical isolate (Figure 1). These results suggested that any of the testicular cells may be responsible for disseminating virus in the infected organ. However, Leydig cells were reportedly less susceptible to ZIKV infection [27], and their role in virus dissemination may be limited. The higher replication levels of ZIKV MR766 could be related to the fact that this strain has been passaged extensively in mouse brains [1,30]. In addition, ZIKV MR766 and ZIKV Paraiba are 89% identical at nucleotide sequence level and 97% (3313/3423) identical at amino acid level (BLAST; https://blast.ncbi.nlm.nih.gov/Blast.cgi). Thus, the ZIKV MR766 strain may have adapted to mammalian cell culture better than the ZIKV Paraiba strain which was passaged only <5 times. However, replication kinetics of ZIKV Paraiba and ZIKV MR766 in Vero E6 cells is similar from 24 to 60 hpi, but ZIKV MR766 replicates to higher titers from 72 to 96 hpi [31]. Therefore, the MR766 replication advantage may be time and cell-type dependent.

Our primary aim was to establish ZIKV persistence in the testicular cell culture systems. All the cells were able to support persistent infection for a few passages, but ZIKV MR766 was more aggressive at killing cells during persistence. ZIKV MR766 persistence in Sertoli cells has been reported for up to 6 weeks [27], but we only observed persistence of this strain for only 4 weeks. This could be a result of the higher ZIKV MR766 titers, which may trigger the host-cell responses leading to cell death. Persistence of ZIKV Paraiba was also limited to a few passages (5 at most in Sertoli and SEM-1 cells; Figure 4). In humans and animals, ZIKV persistence has been reported for time periods longer than the ones we observed in vitro and persistence was mostly of viral RNA in the absence of infectious virus [15,26,32]. In addition, the testes as an organ is immune privileged to protect the organ from highly inflammatory immune reactions [26]. The cells in our study were infected in the absence of the other cells they would normally be associated with in an organ/tissue context, making them more susceptible to rampant virus replication, which kills the cells faster. This notion is supported by a previous report showing that Sertoli cells infected with ZIKV were less adept at mounting an innate immune response in comparison to A549 cells [27].

It is noteworthy that the cell death we observed was slow and progressive until there were not enough cells for continued culture. In vivo, testicular atrophy and associated male infertility has been reported in mice [16,25]. The mechanism of slow cell death we observed in vitro in Hs1.Tes and Sertoli cells persistently infected with ZIKV may be comparable to the mechanism by which testicular atrophy occurs in vivo. Using Sertoli cells, we showed that cell death was via apoptosis because we could detect cleaved caspase 3 by immunofluorescence. Interestingly, not all ZIKV E protein-positive cells were positive for cleaved caspase 3, suggesting that the death signal(s) were not transmitted or activated uniformly in all virus-infected cells. Kumar et al. also showed that the level of apoptosis in Sertoli cells was lower (4–10%) than in A549 cells (58–72%) when infected with ZIKV at 72 hpi [27]. In another study, it was demonstrated that early ZIKV infection was associated with the suppression of cellular growth and proliferation, but antiviral responses were predominate in later stages of infection [28]. However, the specific mechanism which prevents overt apoptosis in testicular cells is yet to be elucidated and this will be an interesting avenue for further investigation using single cell sequencing approaches.

We were puzzled that TCam-2 seminoma cells infected with ZIKV MR766 prevailed up to 7 dpi without CPE, but the cells died upon passage. This intriguing result contrasted with that observed in SEM-1 cells, an intermediate seminoma cells line, which underwent extensive cell death by 7 dpi. These observations supported the hypothesis that ZIKV MR766 was more of an aggressive strain and the phenotype was also dependent on cell type.

We also attempted to comparatively establish a persistent infection of the neuroblastoma cell line (SK-N-SH) cells, but both ZIKV MR766 and ZIKV Paraiba killed the cells leaving no surviving cells. Thus, these observations further indicated that the outcome of ZIKV infection and persistence is cell type dependent.

In summary, we infected several testicular cell lines with ZIKV MR766 and ZIKV Paraiba with the aim of establishing persistent viral infection. The testicular cell lines we used represented germ cells (SEM-1 and TCam-2) and the connective tissue in the form of the fibroblast Hs1.Tes cell line. We also infected germ cell-supporting Sertoli cells, which have been shown to be permissive to ZIKV replication [28,29]. All the cells we tested allowed ZIKV replication and the prototype MR766 strain replicated to higher titers, compared to ZIKV Paraiba. ZIKV Paraiba persisted in Hs1.Tes, TCam-2, SEM-1 and Sertoli cells for up to 5 passages. ZIKV MR766 killed both TCam-2 and SEM-1 seminoma cells but persisted for 3 passages in Hs1.Tes cells and 4 passages in Sertoli cells. Compared to testicular cell lines, the neuroblastoma SK-N-SH cells were killed by both ZIKV strains, thus preventing viral persistence. Our results are consistent with reports that ZIKV persists in testicular cells and suggest that testicular atrophy may be a result of a slow and progressive cell death.
